# P04.05. Wellness versus treatment? Complementary and integrative healthcare (CIH) in the 2007 national health interview survey (NHIS)

**DOI:** 10.1186/1472-6882-12-S1-P275

**Published:** 2012-06-12

**Authors:** B Stussman, L Alekel, R Nahin, E Edwards, P Barnes

**Affiliations:** 1 The National Advisory Council for Complementary and Alternative Medicine, Bethesda, USA; 2 National Center for Health Statistics, Hyattsville, USA

## Purpose

CIH users have been characterized according to the intended use of CIH -- treatment or wellness. This analysis examined reasons adults used specific CIH interventions.

## Methods

Data are based upon the Adult Complementary and Alternative Medicine supplement, the Sample Adult core, and the Family core components of the 2007 NHIS. The survey contained reasons for using CIH including treatment of a specific health problem, general wellness or general disease prevention, and other reasons. We created four mutually exclusive categories for analysis: 1) “yes” to treatment, “no” to wellness; 2) “yes” to wellness, “no” to treatment; 3) “yes” to both; 4) “no” to both. We also examined additional reasons for using CIH.

## Results

More than 50% of adults who used acupuncture, chiropractic or osteopathic manipulation, and biofeedback/hypnosis used it exclusively for treatment, particularly for pain. Vitamin and mineral supplements, diet-based therapies, movement therapies, and yoga, tai chi, and qi gong were most commonly used exclusively for wellness (> 50% of users). Naturopathy and homeopathy were most commonly used for both treatment and wellness (>40% of users). More than 50% of users of non-vitamin non-mineral dietary supplements reported that they used these supplements to improve immune function, to improve physical performance, or because they were recommended by family, friends or co-workers.

## Conclusion

Adults used CIH interventions for a number of reasons that vary by the specific modality. Some interventions were used primarily for treatment and some were used primarily for wellness. Additionally, the intended use varied within the four major CIH categories: natural product therapies, mind-body therapies, manipulative and body-based therapies, and alternative medical systems. Modeling will be used to identify associations between specific CIH therapies and their intended use.

